# Supplementary material for: Raising the bar for patient experience during care transitions in Canada: A repeated cross-sectional survey evaluating a patient-oriented discharge summary at Ontario hospitals
Source: PLoS One. 2022 Oct 4;17(10):e0268418. doi: 10.1371/journal.pone.0268418 (PMC9531793; doi:10.1371/journal.pone.0268418)

**Supplementary Figure 1. GEE model fits with site level data**

*Help Needed After Leaving Hospital*

*
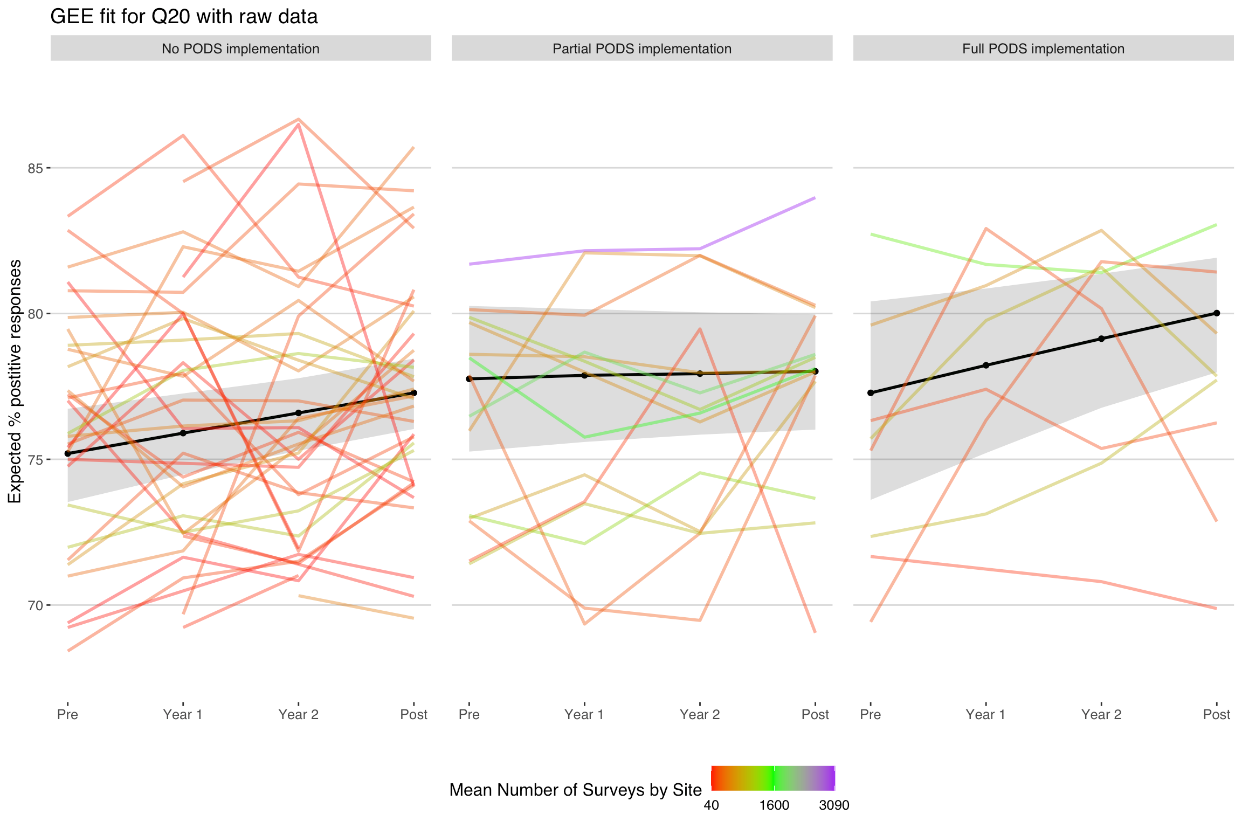
*

*Information in Writing about Symptoms to Look Out For*

*
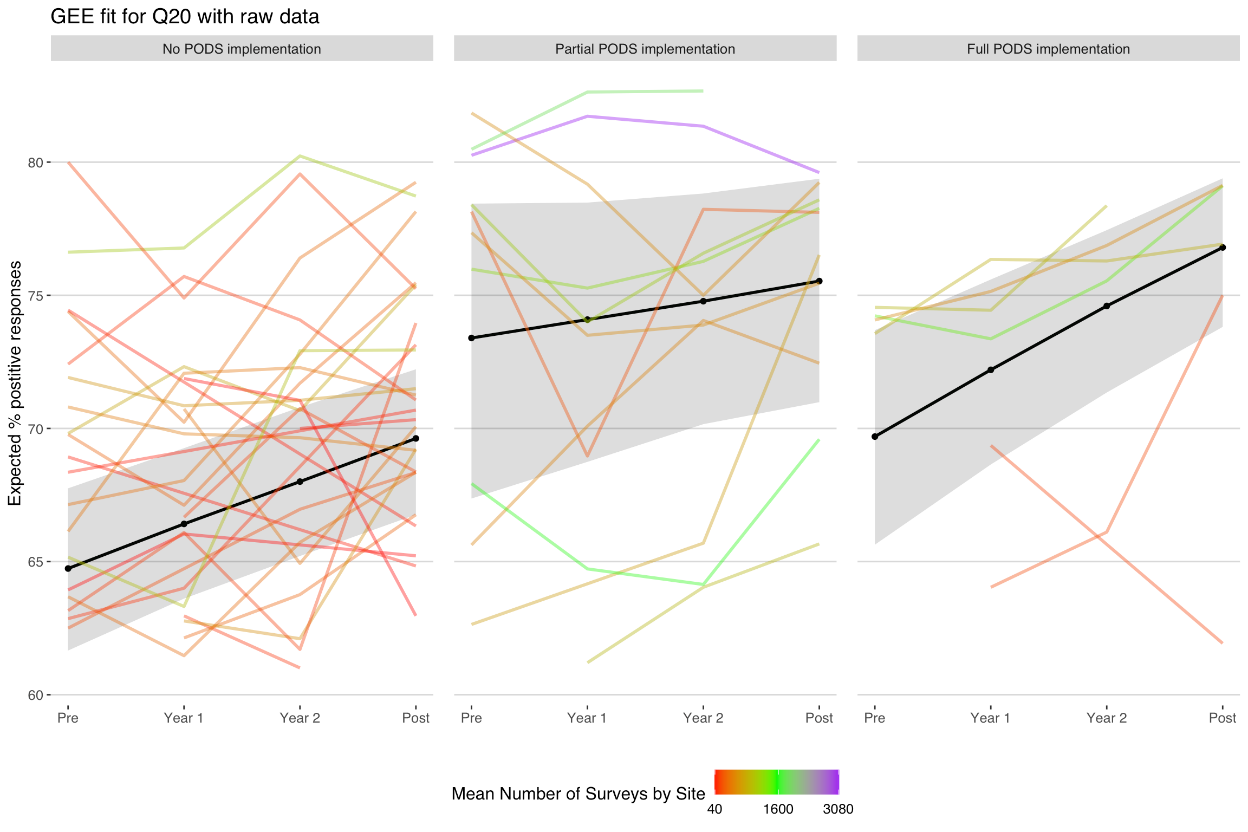
*

*Clear Understanding of Medications*


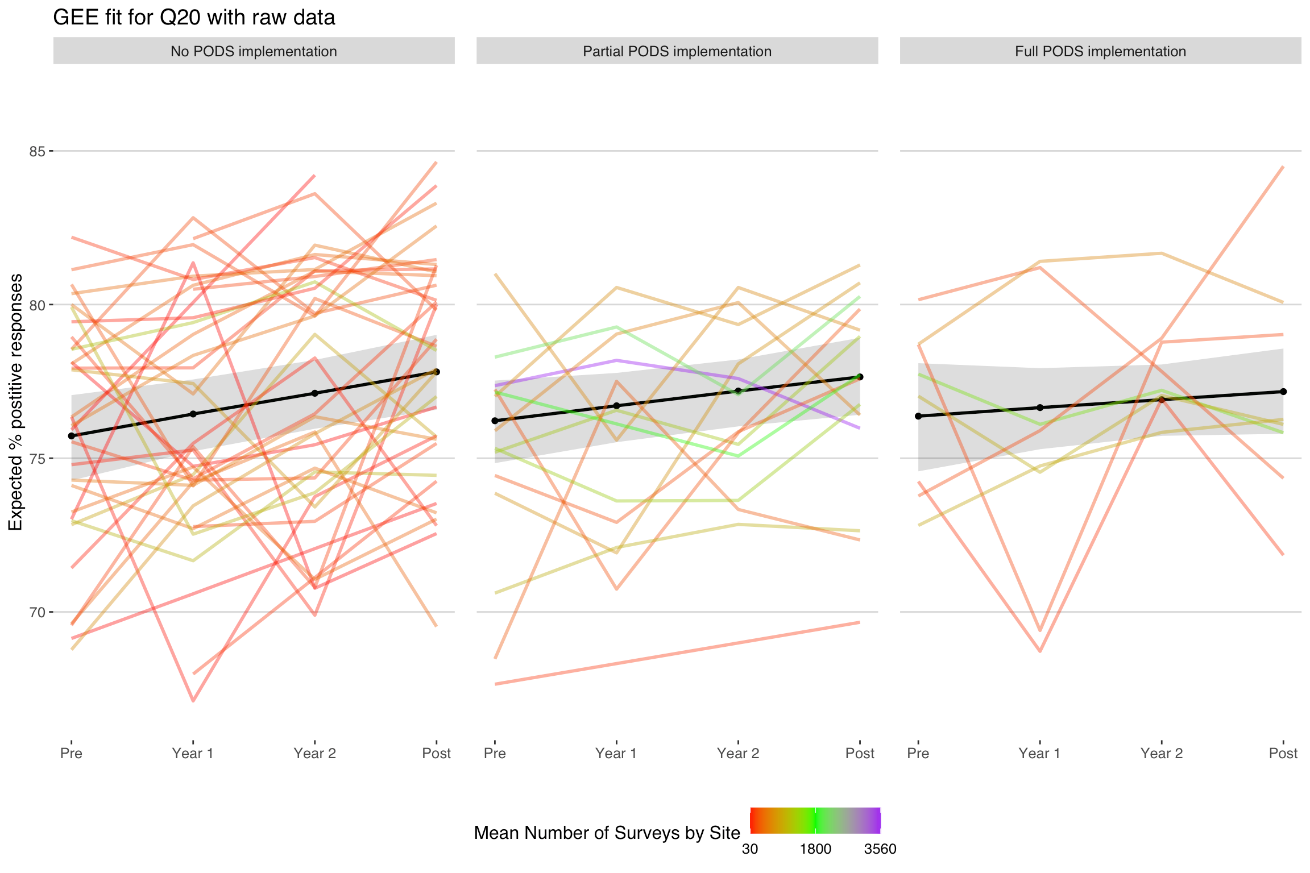


*Information about What to Do if Worried After Leaving Hospital*


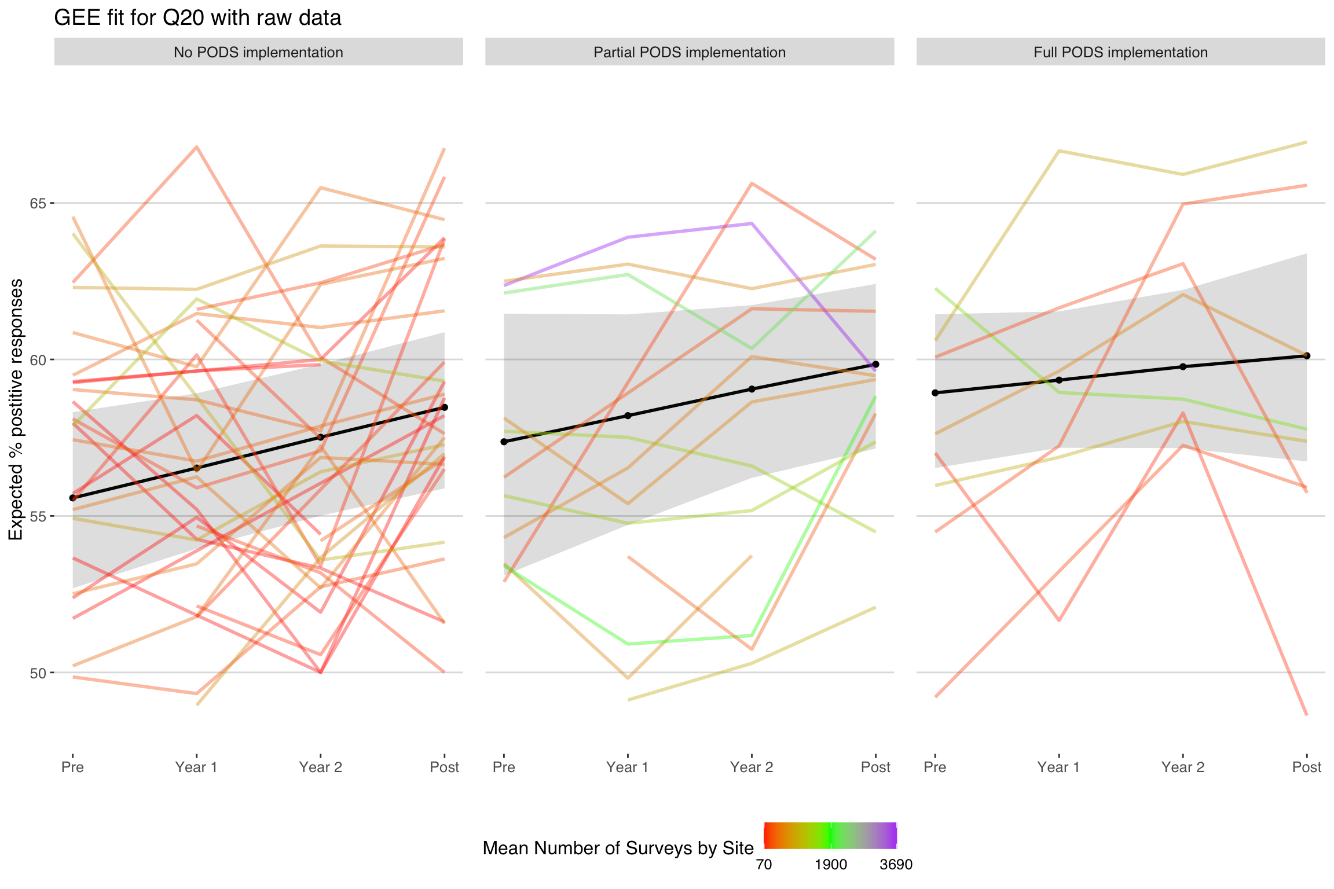

Supplement: S1 Fig — (DOCX) [file pone.0268418.s002.docx]
