# Supplementary material for: Raising the bar for patient experience during care transitions in Canada: A repeated cross-sectional survey evaluating a patient-oriented discharge summary at Ontario hospitals
Source: PLoS One. 2022 Oct 4;17(10):e0268418. doi: 10.1371/journal.pone.0268418 (PMC9531793; doi:10.1371/journal.pone.0268418)
Supplement: Attachment — Submitted filename: Comments.docx [file pone.0268418.s003.docx]

This study adopted a repeated cross-sectional study design (also known as a pseudo-longitudinal design) to assess the impact of a hospital-based care transition intervention (patient-oriented discharge summary; PODS) on patient experience across Ontario acute care hospitals four waves. The authors concluded that the PODS implementation of PODS was associated with higher odds of positive patient experience, particularly with regard to discharge planning. There are several grave issues that should be addressed to further improve on this work.

1. In the main text the declared inferential statistical method was GEE (p. 6). Nonetheless, in the *abstract* it was logistic regression analysis. This is a palpable oversight.
2. With respect to the outcome measures, for the first outcome variable of discharge planning there were two questionnaire items with binary responses (yes and no). As for the other outcome measure (discharge management), the authors employed Likert-type 5-point scale for the first question and 4-point scale for the other question. I am left wondering after reading this piece of text -- how did the authors come up with a single value to indicating “a positive patient experience”?
3. Following on from the above point, I then realized that indeed the authors had analyzed each relevant question separately when I reached Table 3 (starting from the bottom of p. 8). I do not think that this is an appropriate analytical method -- in this study the two outcome variables were discharge planning and discharge management. Questionnaire items are *not* the same as variables. Stated differently, the former is employed to measure the existence or degree of the latter. Indeed, the focus of the authors’ related discussion of analytical results was on the two outcome variables, not each of the four questionnaire items; for example, in the *abstract*, “Interpretation: [*sic*] PODS implementation was associated with higher odds of positive patient experience, particularly for discharge planning [*emphasis added*]. Further efforts should center on discharge management [*emphasis added*]”.
4. P. 6, Involvement of Patient Partners. I do not think the information under this subsection essential at all in the main text. In addition, it is peculiar to present such information *after* the descriptions of statistical analysis.
5. *Abstract*, Results, “512,288 responses were included with mean age 69 ± 14 years (females) and 61 ± 20 years (males).” The authors only presented descriptive statistics as regards the mean ages of both genders, but no information concerning the gender composition of sample patients. Furthermore, although it is not a panel study design, would it be possible that “512,288 responses” included a number of same patients? If yes, then the mean age statistics may not be accurate.
6. Relatedly, with regard to Table 1 on page 7 -- why did the authors kind of *emphasize* age statistics (and age *only*) as to have a table solely for the results?
